# Influence of inter-stimulus interval on 40-Hz auditory steady-state response in patients with schizophrenia

**DOI:** 10.1038/s41537-023-00377-6

**Published:** 2023-07-27

**Authors:** Kang-Min Choi, Chang-Hwan Im, Chaeyeon Yang, Hyun Seo Lee, Sungkean Kim, Seung-Hwan Lee

**Affiliations:** 1 Clinical Emotion and Cognition Research Laboratory, Inje University, Goyang, Republic of Korea; 2 Department of Electronic Engineering, Hanyang University, Seoul, Republic of Korea; 3 Department of Biomedical Engineering, Hanyang University, 222 Wangsimni‑ro, Seongdong‑gu, Seoul, 04763 Republic of Korea; 4 College of Arts and Science, New York University, New York, NY USA; 5 Department of Human-Computer Interaction, Hanyang University, Ansan, Republic of Korea; 6 Department of Psychiatry, Ilsan Paik Hospital, Inje University College of Medicine, Juhwa‑ro 170, Ilsanseo‑Gu, Goyang, 10370 Republic of Korea; 7 Bwave Inc, Juhwa-ro, Goyang, 10380 Republic of Korea

**Keywords:** Schizophrenia, Biomarkers

## Abstract

Decreased 40-Hz auditory steady-state response (ASSR) is believed to reflect abnormal gamma oscillation in patients with schizophrenia (SZ). However, previous studies have reported conflicting results due to variations in inter-stimulus interval (ISI) used. In this study, we aimed to investigate the influence of varying ISI on the 40-Hz ASSR, particularly for patients with SZ and healthy controls (HCs). Twenty-four SZ patients (aged 40.8 ± 13.9 years, male: *n* = 11) and 21 HCs (aged 33.3 ± 11.3 years, male: *n* = 8) were recruited. For every participant, 40-Hz ASSRs were acquired for three different stimulus types: 500, 2000, and 3500 ms of ISIs. Two conventional ASSR measures (total power and inter-trial coherence, ITC) were calculated. Several additional ASSR measures were also analyzed: (i) ISI-dependent power; (ii) power onset slope; (iii) power centroid latency; (iv) ISI-dependent ITC; (v) ITC onset slope (500, 2000, 3500 ms); (vi) ITC centroid latency (500, 2000, 3500 ms). As ISI increased, total power and ITC increased in patients with SZ but decreased in HCs. In addition, patients with SZ showed higher ISI-dependent ITC, which was positively correlated with the psychotic symptom severity. The abnormal ITC onset slope and centroid latency for the ISI-500 ms condition were associated with cognitive speed decline in patients with SZ. Our study confirmed that the 40-Hz ASSR could be severely influenced by ISI. Furthermore, our results showed that the additional ASSR measures (ISI-dependent ITC, ITC onset slope, ITC centroid latency) could represent psychotic symptom severity or impairment in cognitive function in patients with SZ.

## Introduction

Gamma band oscillations have been widely believed to be associated with a variety of cognitive processes. It is known to help establish temporally precise synchronization in local cortical networks^1,2^. Furthermore, numerous electroencephalography (EEG) studies have reported that gamma-band oscillations play an essential role in various high-level cognitive functions, such as perceptual integration, attentional selection, and memory operation^3,4^. Based on these findings, several pathophysiological studies have paid attention to the relationship between gamma band malfunction and the characteristics of psychiatric disorders.

The gamma band malfunction has been regarded as a critical characteristic, in particular, for schizophrenia (SZ). The gamma band abnormality is associated with abnormalities in fast-spiking parvalbumin-expressing cells and hypofunction of the N-methyl-D-aspartate (NMDA) receptor in patients with SZ^5,6^. The gamma band malfunction of SZ has been investigated based on EEG using various experimental paradigms, such as resting-state, oddball paradigm, N-back, and 40-Hz auditory steady-state response (ASSR)^7^. Among them, 40-Hz ASSR has been widely used for the evaluation of gamma band abnormality in patients with SZ, with its simplicity of experimental designs and high test-retest reliability^8–10^.

Although it is generally believed that SZ is characterized by the decreased 40-Hz ASSR based on the biological establishment^11^, several studies observed no significant difference^12,13^ and others even reported an increase in ASSR among patients with SZ^14,15^. These conflicting results may stem from different experimental paradigms. ASSR is thought to be affected by various factors of auditory stimulation such as stimulus type or duration^16,17^. Furthermore, our previous study showed that inter-stimulus interval (ISI) could also be an important factor potentially influencing ASSR^15^. Interestingly, several studies using relatively longer ISIs (>3 s) reported no decrease in the ASSR measures in patients with SZ^12–15^. In comparison, a majority of studies using shorter ISIs (<1 s) reported a decrease in the ASSR measures in SZ^11,18,19^. Nonetheless, the influence of ISI on the ASSR is yet to be probed in patients with SZ.

ASSR measures are thought to be associated with cognitive deterioration and symptom severity in patients with SZ^11^. Several studies have demonstrated the association between ASSR measures and clinical symptoms in SZ. For example, Spencer et al. ^18^ found a positive correlation between 40-Hz ASSR phase synchrony in the left hemisphere and hallucination symptoms in patients with SZ. Koshiyama et al. ^20^ presented that 40-Hz ASSR power could be a prognostic factor of psychotic symptoms in patients with SZ. Although recent studies reported the association between the conventional ASSR measures and cognitive decline^15,20,21^, large variabilities in findings across these experiments have been reported. A few studies have recently focused on developing novel ASSR measures, considering more pathophysiological characteristics. For example, Roach et al. ^19^ developed the phase-locking angle based on the finding that patients with SZ showed relatively late phase synchronization to the 40-Hz auditory steady-state stimulation^22^. The phase-locking angle was associated with psychotic symptom severity in patients with SZ^23^. Thus, considering ASSR for various phases of the stimulation period has repeatedly been advocated as a significant contributor to advancing our understanding of the pathophysiology of SZ within the same experimental paradigm.

In this study, we aimed to explore the alteration of ASSR measures between SZ and HC groups along different ISIs. The study involved the auditory stimuli paradigms with three different ISIs (500, 2000, 3500 ms) presented to both groups. Furthermore, we also aimed to demonstrate our hypothesis and the importance of the time period with respect to the stimulation period associated with psychotic symptoms or reduced cognitive or social function. To the best of our knowledge, this is the first study examining the effects of ISIs on the 40-Hz ASSR measures and highlighting the importance of the temporal characteristics of ASSR using mathematically developed measures.

## Method

### Participants

A total of 24 patients with SZ (40.8 ± 13.9 years, Male: *n* = 11) and 21 HCs (33.7 ± 11.5 years, Male: *n* = 8) were enrolled in the study. Patients with SZ were recruited from the Department of Psychiatry at the Inje University Ilsan Paik Hospital. The diagnosis of SZ was based on the Structured Clinical Interview for the Diagnostic and Statistical Manual of Mental Disorders, 4th edition (SCID)^24^. The patients did not have any neurological illness, mental retardation, substance abuse, head injury, or impaired hearing ability. HCs were recruited from the community using flyers and posters. They were required to have no history of head injury, medications with psychiatric disorders, and also have no family history of psychiatric disorders. No handedness information was obtained from either patients or HCs. All the participants signed an informed consent form approved by the Institutional Review Board at Inje University Ilsan Paik Hospital before participating (IRB No. 2015-07-044).

### Psychotic symptom and socio-cognitive function

Psychotic symptoms were assessed using the Positive and Negative Syndrome Scale (PANSS)^25^ and the Clinical Assessment Interview for Negative Symptoms (CAINS)^26^. Cognitive function was assessed by Digit-Symbol Substitution Test (DSST)^27^ and Digit Span Test (DST)^28^. Moreover, a social function was assessed by the Social and Occupational Functioning Assessment Scale (SOFAS)^29^. For more detail, see supplementary material.

### Auditory stimuli presentation

The auditory stimuli were presented through noise-canceling MDR-D777 headphones (Sony, Tokyo, Japan), binaurally. During the stimulus presentation, participants were instructed to gaze at the fixation cross on the monitor, while seated in a comfortable chair. For every participant, three types of auditory stimuli were presented, the order of which was randomly assigned. Their ISIs were 500, 2000, and 3500 ms, respectively. All the other conditions of the stimuli were identically set: 1 ms of click sound (80 dB) trains at 40-Hz were presented during 500 ms. Each type of stimulus was presented 150 times consecutively in a single session. The stimuli were presented by E-prime software (Psychology Software Tools, Pittsburgh, PA, USA).

### Signal acquisition and pre-processing

The EEG signal was acquired with 64 Ag/AgCl electrodes using the SynAmps amplifier (Neuroscan, Compumedics USA, Charlotte, NC, USA) mounted on NeuroScan Quik-cap according to the extended 10–20 system, with synchronized to auditory stimulus presentation onset by E-Prime. The additional electrodes were attached to acquire vertical and horizontal electrooculogram (EOG) signals, positioned above and below the left eye and outer canthus of both eyes, respectively. All signals were recorded with a 0.1–100 Hz band pass filter and a 60 Hz notch filter, with a sampling rate set at 1000 Hz. The impedance of each electrode was maintained below 5–kΩ during the whole experimental period. The ground and reference electrodes were positioned on the forehead and both mastoids, respectively.

The recorded EEG signals were preprocessed with CURRY 7 (Compumedics USA, Charlotte, NC, USA) software. The acquired signals were manually inspected to reject bad blocks by a trained person without any prior information about the data origin. Eye movement and eye blinking artifacts were removed using a mathematical procedure relying on the covariance and regression method implemented in CURRY 7^30^. Subsequently, the EEG data were segmented from the 300 ms pre-stimulus to the 1000 ms post-stimulus. The 6th-order Butterworth band-pass filtering was implemented with a cutoff frequency of 1–90 Hz. The baseline was corrected using the pre-stimulus period. Finally, the epochs with amplitudes that exceeded ±90 µV over any EEG electrodes were excluded for further analysis. For each participant, at least 50 epochs were included for further analysis for every stimulus type.

### Time-frequency analysis

The overall data analysis was performed on MATLAB R2019b (MathWorks; Natick, MA, USA). To evaluate the spectral power and the phase similarity of oscillatory activity due to the auditory stimuli across all trials, two types of conventional measures consisting of the total power and inter-trial phase coherence (ITC) were calculated, respectively. The calculation was performed using an implemented function in the EEGLAB toolbox^31^. Furthermore, six additional ASSR-related measures were also assessed with two perspectives: (i) differentiation of ASSR trend by ISIs; (ii) temporal characteristics of auditory response under each ISI condition. All the ASSR measures were calculated at the CZ channel to minimize laterality effect on the ASSR. The phase-locking angle was also analyzed for all ISI conditions (see details in Supplementary material).

#### Conventional 40-Hz ASSR measures: total power and ITC

To evaluate the total power, event-related spectral perturbation (ERSP) was calculated using a short-time Fourier transform for every 5 ms of time bin using a Hanning window size of 250 ms; hence, time points ranged from −175 to 875 ms (Table 1). To evaluate the ITC, phase synchronization of oscillatory activity was calculated across every trial. These two types of time-frequency analysis-based conventional ASSR measures were calculated for each type of ISI (i.e., 500, 2000, 3500 ms). All the conventional ASSR measures were calculated as averaged values at the CZ channel while the stimulus presented (i.e., 0–500 ms), for 36–45 Hz^15,32,33^. Please refer to the supplementary material for the detailed calculation process.

**Table 1:** 40-Hz ASSR measures in the study.

ASSR measures	Power	Phase
Conventional measures (500, 2000, 3500 ms)	Total power	ITC
Additional measures		
ISI dependence	ISI-dependent power	ISI-dependent ITC
Onset slope (500, 2000, 3500 ms)	Power onset slope	ITC onset slope
Centroid latency (500, 2000, 3500 ms)	Power centroid latency	ITC centroid latency

Two conventional measures, total power and ITC were calculated. Based on them, six additional measures were additionally calculated.

#### Additional 40-Hz ASSR measures: ISI dependence, onset slope, centroid latency

ISI-dependent power was calculated from the total power to assess the changing pattern of the total power in proportion to increasing ISI conditions (Table 1). It was developed to assess the effects of the ISIs based on our main hypothesis. The ISI-dependent power was estimated by the slope of the total power in relation to the ISI conditions using a linear regression model. In addition, two more types of power-related measures were designed to assess the importance of the time period for the stimulus for each ISI condition (i.e., 500, 2000, 3500 ms): power onset slope and power centroid latency. Specifically, three power onset slopes were calculated to assess the steepness of the initial response to the stimulus for each ISI condition. The power onset slope was developed according to the widely accepted phenomenon of increasing power observed in the initial period (i.e., ~ 100 ms) in patients with SZ compared to HCs^19,22,23,34^. The early period was assumed to last until 100 ms from the stimulus onset in the studies (Supplementary Material)^13,34,35^. Three power centroid latencies were calculated to assess comprehensive dynamic temporal characteristics for each ISI condition. The power centroid latency was developed by findings of prolonged peak latency in patients with SZ^36–39^. We assumed that ASSR may likewise be represented by a certain temporal latency. In the current study, the power centroid latency was defined as a center of gravity as a function of time.

From the ITC, additional three types of ASSR measures were also computed. In consequence, ISI-dependent ITC was calculated according to the increasing ISI conditions. Three ITC onset slopes and three ITC centroid latencies were calculated for each ISI condition (500, 2000, 3500 ms). The detailed information including mathematical expressions of the additional ASSR measures are delineated in the supplementary material (Supplementary Table S1).

### Statistical analysis

To verify the assumption of data normality, skewness and kurtosis were examined for data distribution, including demographic data and ASSR measures. Normal distribution was assumed given that all the absolute values of skewness and kurtosis were less than 2 and 7, respectively^40^. A statistical power analysis confirmed that our sample size was sufficient in the study (Supplementary Material)^41^.

For comparison of group differences in age and education, a two-tailed Student’s *t* test was used. For the comparison of the sex ratio, the chi-square test was used. To evaluate the group-by-ISI interaction, repeated-measures analysis of variance (rmANOVA) was performed: three types of ISIs (i.e., 500, 2000, 3500 ms) as within-subjects factors and the group (SZ vs. HC) as the between-subjects factors. Education level was used as a covariate. Regarding rmANOVA, when Mauchly’s sphericity assumption was not met, the Greenhouse-Geisser correction was used. If significant group-related interaction was observed, post-hoc analyses were performed in two different ways; an independent t-test and the rmANOVA for each group. In order to adjust for multiple comparisons, the Bonferroni correction was employed.

The associations between both conventional and additional ASSR measures and psychotic symptom severity and socio-cognitive function were examined using Pearson correlation analysis with a bootstrap resampling technique (*n* = 5000) to avoid multiple correction issues. All the statistical analyses were performed using SPSS 27 (SPSS, Inc., Chicago, IL, USA).

## Results

### Demographic, psychological characteristics, and epoch used

There were no significant differences between SZ and HC groups in terms of age and sex (*p* = 0.069 and *p* = 0.764, respectively); however, there was a significant group difference in education years (*p* = 0.001; Table 2). Hence, the effect of education was controlled for all group comparison tests. All the cognitive function was significantly lower in patients with SZ (*p* < 0.01). There was no significant group-by-ISI interaction in the number of epochs used in the analysis in the groups and ISIs (*p* = 0.977).

**Table 2:** Demography data.

	SZ (*n* = 24)	HC (*n* = 21)	*p*
Age (years)	40.8 ± 13.9	33.7 ± 11.5	0.069
Sex	M 11 / F 13	M 8 / F 13	0.764
Education (years)	12.42 ± 2.87	15.19 ± 2.44	0.001*
DSST	7.61 ± 3.82	12.22 ± 2.90	<0.001*
DST			
Forward	8.96 ± 3.42	11.89 ± 3.27	0.008*
Backward	8.09 ± 2.09	13.11 ± 3.58	<0.001*
DOI	14.51 ± 14.65		
Dosage (mg)			
Antipsychotics (Cpz-equivalent)	521.11 ± 1075.12		
PANSS			
Positive	13.71 ± 4.31		
Negative	15.83 ± 5.40		
General	31.58 ± 6.85		
Total	61.13 ± 14.51		
SOFAS	66.71 ± 11.15		
CAINS			
Map	17.48 ± 8.31		
Exp	6.70 ± 3.75		
Total	24.17 ± 11.01		
Epoch			
ISI 500	103.17 ± 21.69	109.19 ± 23.82	0.977
ISI 2000	101.83 ± 23.17	108.95 ± 23.83	
ISI 3500	102.17 ± 20.49	105.48 ± 22.70	

For all the further group comparison analysis, education level was controlled because there was a significant group difference in education level.

*SZ* schizophrenia, *HC* healthy control, *DSST* Digit-Symbol Substitution Test, *DST* Digit Span Test, *DOI* duration of illness, *PANSS* Positive and Negative Syndrome Scale, *Cpz* Chlorpromazine, *SOFAS* Social and Occupational Functioning Assessment Scale, *CAINS* Clinical Assessment Interview for Negative Symptoms, *Map* motivation and pleasure, *Exp* expression, *ISI* inter-stimulus interval, *Epoch* the number of epochs used for analysis.

*p < 0.05.

### Conventional 40-Hz ASSR measures

**Fig. 1:**
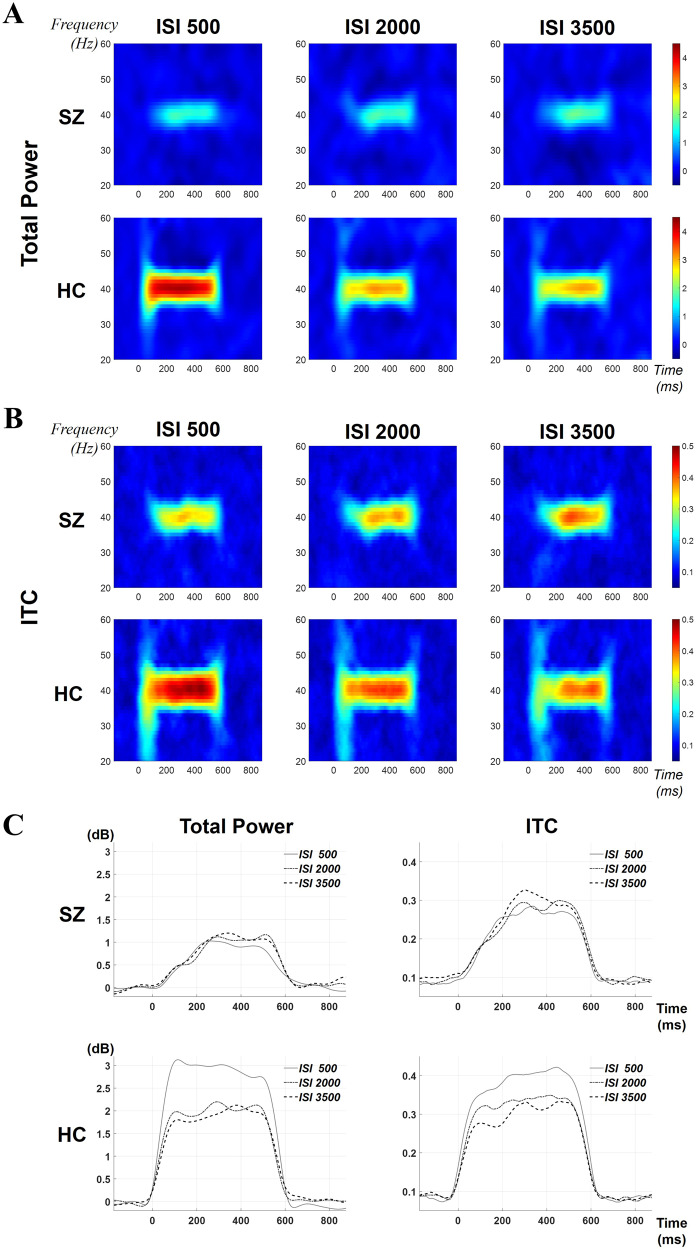
Grand averaged time-frequency maps and time-courses of total power and ITC for patients with SZ and HCs. **A** Time-frequency maps of total power. **B** Time-frequency maps of ITC. **C** Time-courses of total power and ITC. ISI inter-stimulus interval, SZ schizophrenia, HC healthy control, ITC inter-trial coherence.

Overall, SZ showed a gradual increase in both conventional ASSR measures as ISIs increased. However, the patterns were reversed in HC (Fig. 1A, B). In addition, patients with SZ showed relatively late ASSR to the auditory stimulus (i.e., slow increases of total power and ITC), particularly for the stimulus onset period (0–200 ms, Fig. 1C).

**Fig. 2:**
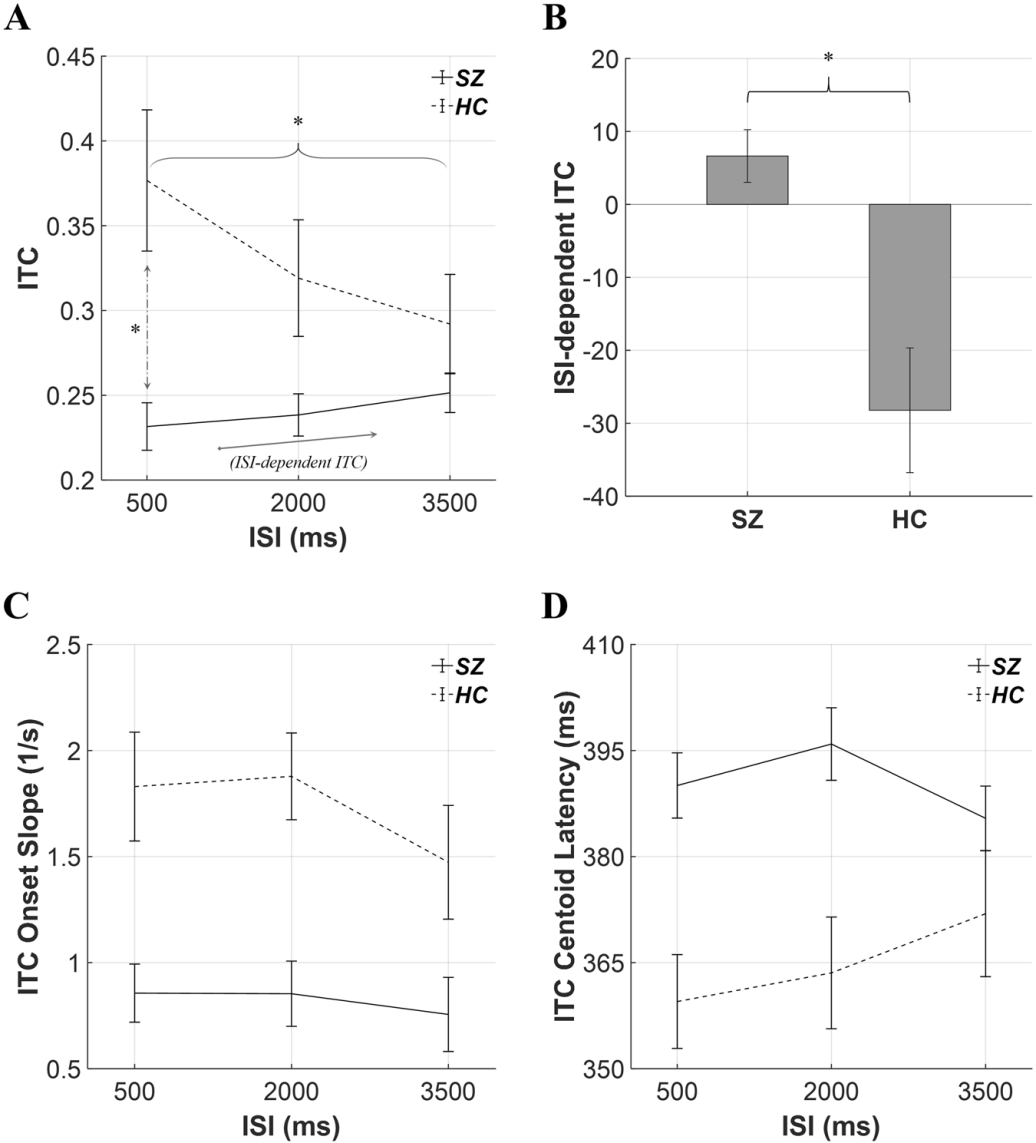
Comparison of ITC-related ASSR measures. **A** Grand averaged ITCs for each ISI condition. There was a marginally significant group-by-ISI effect. The post-hoc analysis revealed that patients with SZ showed significantly lower ITC with ISI-500 ms condition. A slope of ITC as a function of ISI was evaluated as ISI-dependent ITC. **B** Grand averaged ISI-dependent ITC. **C** Grand averaged ITC onset slope. There was a significant group effect (SZ < HC). **D** Grand averaged ITC centroid latency. There was a significant group effect (SZ > HC). The error bars indicate standard errors. SZ schizophrenia, HC healthy control, ITC inter-trial coherence, ISI inter-stimulus interval, *p < 0.05.

There were no group-related interactions or effects for total power. For ITC, a marginally significant group-by-ISI interaction was observed (*p* = 0.091, Fig. 2A). An independent t-test was followed as a post-hoc analysis and revealed that SZ showed reduced ITC compared with HC in ISI-500 ms condition (SZ vs. HC = 0.23 vs. 0.38; *p* = 0.024), and each group rmANOVA showed that only HC showed significantly decreased ITCs as ISIs increased (*p* = 0.020). For HC, ITC with ISI-500 ms condition was greater than with ISI-3500 ms condition (ISI-500 vs. ISI-3500 = 0.38 vs. 0.29; *p* = 0.011). There was no other significant group-related interaction.

### Additional ASSR measures

For the total power-derived ASSR measures, neither group-related interaction nor group effect showed statistical significance.

For the ITC-derived measures, there was a significant group difference in ISI-dependent ITC (SZ vs. HC = 6.62 vs. −28.22; *p* = 0.016; Fig. 2B). For the ITC onset slope, a significant group effect was observed (SZ vs. HC = 0.82 1/s vs. 1.73 1/s; *p* = 0.009; Fig. 2C). For the ITC centroid latency, a significant group effect was observed (SZ vs. HC = 390.46 ms vs. 364.99 ms; *p* = 0.019; Fig. 2D). There was no other significant group-related interaction or group effect.

### Correlation analysis

Because both the duration of illness and dosage of antipsychotics showed no significant correlation with all the ASSR measures and psychological measures, these variables were not considered as covariates. The conventional ASSR measures did not show any significant correlations.

**Fig. 3:**
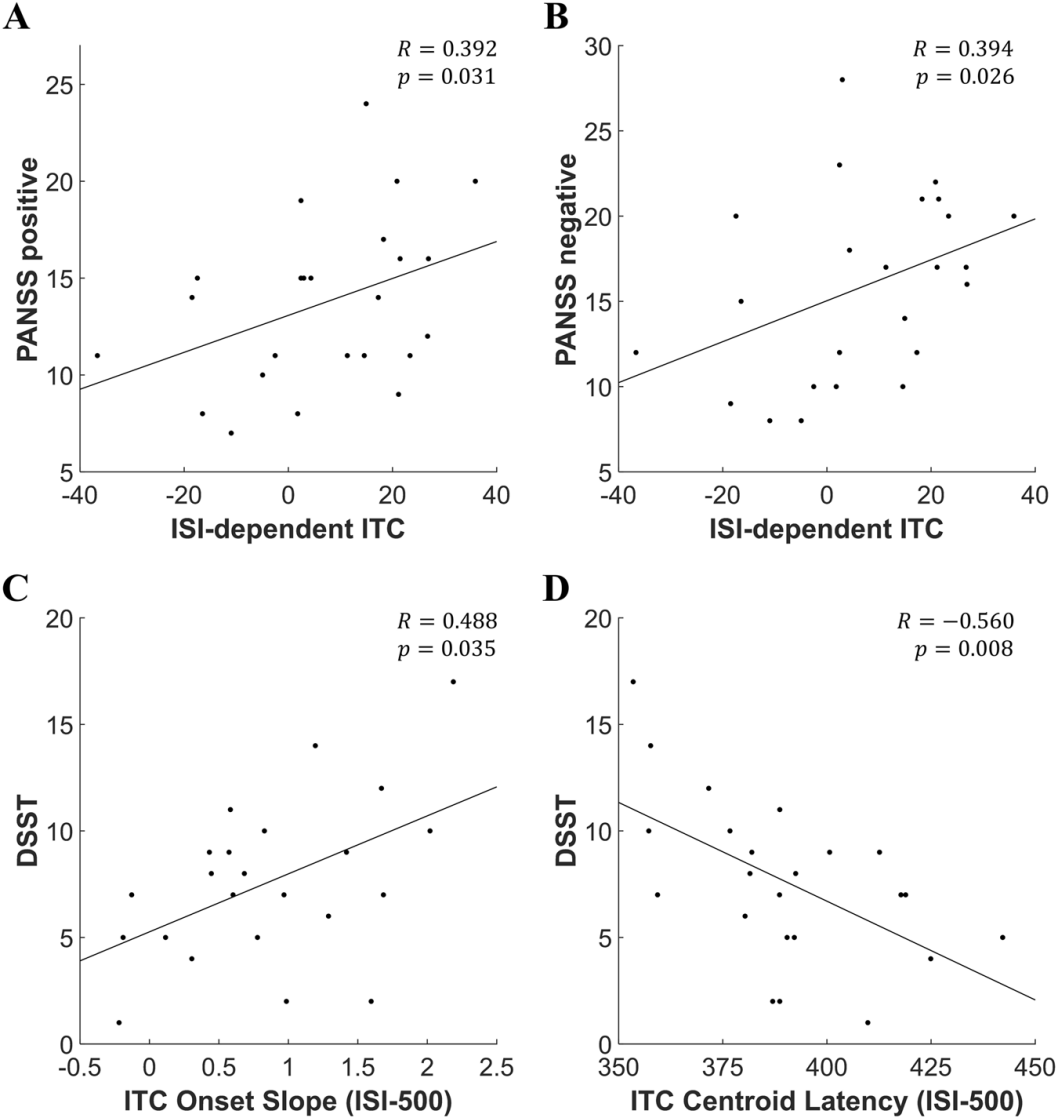
Results of correlation analysis in patients with SZ. **A**, **B** ISI-dependent ITC was positively correlated to both the PANSS positive and negative. **C**, **D** ITC onset slope and ITC centroid latency with ISI-500 condition were positively and negatively correlated to the DSST, respectively. SZ schizophrenia, ISI inter-stimulus interval, ITC inter-trial coherence, ISI-dependent ITC ISI dependence of ITC, DSST digit-symbol substitution test.

For the additional ASSR measures, the ISI-dependent ITC showed significant positive correlations with both the PANSS positive (r = 0.392, *p* = 0.031, 95% CI 0.025–0.674; Fig. 3A) and negative scale (r = 0.394, *p* = 0.026, 95% CI 0.060–0.703; Fig. 3B). The ITC onset slope for the ISI-500 ms condition showed significant correlations with the DSST in SZ (r = 0.488, *p* = 0.035, 95% CI 0.041–0.765; Fig. 3C). The ITC centroid latency with ISI-500 ms condition showed significant correlations with the DSST in SZ (r = −0.560, *p* = 0.008, 95% CI −0.757–−0.236; Fig. 3D). However, in ISI-2000 and 3500 ms conditions, the ITC onset slope and the ITC centroid latency did not show any significant correlations with psychological measures. In addition, no significant correlations were found between the total power-derived additional measures and the psychological measures.

## Discussion

In the present study, we revealed that ISI is an essential factor in the 40-Hz ASSR. Importantly, our results demonstrated that ISI could differently influence ASSR measures between SZ and HC. As ISIs increased, both total power and ITC showed an increasing tendency in patients with SZ but a decreasing trend in HCs, which may subserve to clarify inconsistencies in findings. Interestingly, patients with SZ showed higher ISI-dependent ITC, which was positively associated with positive and negative psychotic symptom severity. In addition, patients with SZ showed abnormal ITC onset slope and centroid latency, particularly for the short ISI condition (500 ms), which was associated with cognitive processing speed decline.

### Effects of ISIs on ASSR

Both total power and ITCs exhibited a similar response tendency to 40-Hz auditory stimuli with three different ISIs. Both measures showed a decreased tendency according to increasing ISI condition in HC, however, an increased tendency according to increasing ISI condition in SZ.

As aforementioned, the effects of ISIs on ASSRs remain unexplored and warrant further investigation. For a comprehensive understanding of our experimental outcomes, additional neurobiological research is crucially required. However, prior auditory event-related potential (ERP) studies concerning the effects of ISIs may provide valuable insights. Particularly, two auditory ERP models could explain our ISI effects. The first one is a refractoriness model. In the simple monotonous auditory ERP studies, it is generally shown that the longer the ISIs, the larger the peak amplitude^42–45^. Recent studies have conceptualized this phenomenon as a refractoriness model^44^, hypothesizing that auditory response recovery takes time due to the refractoriness period of the neural generator. Importantly, this model seems to be particularly influential in patients with SZ, as the patients are known to have an extended refractory period compared to HC^46,47^. In other words, a 500 ms ISI might not be enough for the neural generators to recover in patients with SZ. In fact, patients with SZ showed significantly lower ITC for the ISI-500 ms condition only.

The second one is a cognitive capacity model. In deviant stimuli-related (mismatch negativity or Go/Nogo) auditory ERP paradigms, ERP components due to the deviant stimulus were generally decreased^48–50^ and reaction time increased^49^ as ISIs increased for HCs. Importantly, this model seems to be particularly influential in HCs as the decrease in ERP components was found to be insensitive in patients with SZ, reflecting a deficit in cognitive capacity in these patients^51^. The cognitive capacity model is established by the neurobiological mechanisms: both the abnormalities in the deviant-ERP and ASSR in patients with SZ are believed to originate from dysfunctions in the NMDA receptors^5,6,52^, linked to cognitive decline^53^. The cognitive capacity model might show neurophysiological relation to the sensory gating theory. Repeated and identical external stimuli were expected to cause attenuated responses during neurophysiological information processing in HC. However, patients with SZ, who tend to show sensory gating deficits, showed no attenuated response to repetitive stimuli^54^.

### Relationship between 40-Hz ASSR and psychological measures

The conventional ASSR measures did not reflect SZ-specific pathophysiology. However, the additional measures significantly reflect the symptom severity and cognitive pathology of SZ. In our results, ISI-dependent ITC was positively associated with the psychotic symptom severity and ITC onset slope and centroid latency for ISI-500 ms condition were associated with the cognitive processing speed.

#### ISI-dependent ITC

ISI-dependent ITC assessed the change in the pattern of ITC by an increase in ISI condition. The opposite tendency was observed between SZ (increasing) and HC (decreasing). ISI-dependent ITC was positively correlated with the PANSS positive and negative scores, highlighting the study’s central hypothesis that the effect of ISIs on the ASSR measures was linked to the pathophysiological characteristics of SZ. Several replication studies found a relationship between the conventional ASSR measures and psychotic symptoms in SZ. Some of them found an association with the positive symptoms, particularly for the auditory hallucinatory behaviors^55,56^, whereas others found that with the negative symptoms^57^. In our study, ISI-dependent ITC showed a positive correlation with both PANSS positive and negative but none of the conventional ASSR measures correlated with psychotic symptom severity for all the ISI conditions. Our results suggest that ISI-dependent ITC could be a more sensitive measure to evaluate the psychotic symptom severity in patients with SZ.

#### ITC onset slope

ITC onset slope assessed the steepness of the initial response to the stimulus according to increasing ISI condition. ITC onset slope was significantly higher in HC compared to SZ. Our results are in line with the previous pathophysiological finding, the impaired dynamic perceptual switching in patients with SZ^57^. Lower ITC onset slope in patients with SZ was linked to the delayed phase synchrony to the external stimuli, accounting for their relatively slow or insensitive responses to the stimuli^58^. This pathophysiological characteristic could impair rapid temporal synchronization across cortical networks, causing cognitive processing speed dysfunction. In fact, the ITC onset slope with ISI-500 ms was positively correlated with the declined working memory speed in patients with SZ, underpinning our suggestion.

#### ITC centroid latency

ITC centroid latency assessed comprehensive dynamic temporal characteristics according to increasing ISI conditions. ITC centroid latency was significantly longer in SZ in comparison to HC. Further, ITC centroid latency in SZ negatively correlated with the DSST only in the ISI-500 ms condition. Our results conclusively propose that shorter ISI condition serves as a better assessment tool for examining the cognitive decline in patients with SZ. This outcome might be attributed to the property of ITC centroid latency, reflecting various stimulus-driven latent information about the phase synchronization, such as initial-, ongoing-, and post-stimulus phases. Importantly, both the ITC onset slope and centroid latency can be readily calculated within the commonly used ASSR paradigm, specifically in the ISI-500 ms condition.

### Limitations

There are several limitations to the study. First, there was a discrepancy in education levels between SZ and HC. Therefore, all the statistical models for group comparisons controlled for the education levels as a covariate. Second, patients with SZ were not in a drug-naive condition. However, the dosage of antipsychotics and the duration of illness were not correlated to the ASSR and psychological measures in the study. Third, more replication studies with large sample size are required to render our experimental results more generalizable.

## Conclusion

In the present study, we investigated the effects of ISIs on the 40-Hz ASSR between patients with SZ and HCs. The conventional ASSR measures gradually increased in SZ but decreased in HC as ISIs increased, suggesting that ISI is an important factor influencing 40-Hz ASSR. Our experimental results also showed that the additional ASSR-related measures may serve as biomarkers of psychotic symptom severity and cognitive decline in patients with SZ. We anticipate that ASSR measures that consider the neurophysiological characteristics more comprehensively will have a greater potential for predicting psychological measures in patients with SZ, as well as for clinically discriminating between SZ and HC.

## Supplementary information

Supplementary material (rev)
