# Supplementary material for: Influence of inter-stimulus interval on 40-Hz auditory steady-state response in patients with schizophrenia
Source: Schizophrenia (Heidelb). 2023 Jul 27;9(1):46. doi: 10.1038/s41537-023-00377-6 (PMC10374560; doi:10.1038/s41537-023-00377-6)
Supplement: Supplementary file 1 — Supplementary material (rev) [file 41537_2023_377_MOESM1_ESM.docx]

**Supplementary materials**

Contents

1. Details about the socio-cognitive function assessment
2. Details about the conventional 40-Hz ASSR measures
3. Details about the additional 40-Hz ASSR measures
   1. Power onset slope
   2. Power centroid latency
   3. Mathematical expressions of the additional ASSR measures
   4. Phase-locking angle
4. Statistical power analysis
5. Supplementary results: Phase-locking angle
6. References

**1. Details about the socio-cognitive function assessment**

For the assessment of cognitive function, the Digit-Symbol Substitution Test (DSST) and Digit Span Test (DST) were performed. The DSST, a subtest for the Wechsler Intelligence Scale, was used to assess psychomotor and processing speed as well as visual and working memory. The total scores were calculated as the total number of correctly drawn symbols, suggesting that higher scores mean highly effective performance. The DST was used to assess attentional capacity and working memory. It was composed of two tests: Digit Span Forward (DSF) and Digit Span Backward (DSB). The overall scores were the sum of the number of correct answers in the forward and backward digit span, with greater scores indicating better performance.

For the assessment of social function, the Social and Occupational Functioning Assessment Scale (SOFAS) was acquired. The SOFAS was conducted to measure a level of social, occupational, and interpersonal functioning. It is a clinician-rated one-item scale based on the Diagnostic and Statistical Manual for Mental Disorders 4^th^ Edition (1). The range of the score varies from 0 to 100, and a greater level of score indicates greater social functioning (2).

**2. Details about the conventional 40-Hz ASSR measures**

For the evaluation of the total power and inter-trial phase coherence (ITC), event-related spectral perturbation (ERSP) was calculated. The ERSP was calculated with a short-time Fourier transform for every 5 ms of time bin using a Hanning window size of 250 ms, using ‘newtimef’ function implemented in the EEGLAB toolbox.

For evaluation of the total power, the power spectrum was normalized to the average power of the baseline period (i.e., before 0 ms) to measure the power spectral differences caused by the auditory stimulus presentation for every trial. The normalized power spectra for each stimulus were then averaged over trials. Consequently, the resultant total power for each participant was calculated for every ISI condition (i.e., 500, 2000, 3500 ms). The calculated power values were then transformed to the decibel (10 log_10_) (3). No additional smoothing or filtering process was applied in the calculation.

For evaluation of the ITC, phase synchronization of oscillatory activity was calculated across every trial. This quantification was conducted by dividing the complex values of the ERSP by its absolute values (i.e., complex norm) for every time point (i.e., 5 ms), to cancel out the influence of the signal amplitude. Hence, the ITC values range from 0 (totally non-phase-locked at a time point across trials) to 1 (fully phase-locked). The resultant ITC for each participant was also calculated for every ISI condition.

**3. Details about the additional 40-Hz ASSR measures**

The 40-Hz ASSR has been proposed to be decomposed into two periods: an initial period, lasting from 0 to 100 ms, and a steady-state period, lasting from 200 to 500 ms (4, 5, 6, 7). Despite the significance being repeatedly reported (4, 5, 8, 9), a majority of ASSR studies have still not still focused on the temporal characteristics in 40-Hz ASSR. Nonetheless, the diverse time courses of the ASSR could provide substantial insights into the abnormal neural circuits in patients with SZ. In this study, we developed several mathematical ASSR measures to underscore the importance of the temporal characteristics of the 40-Hz ASSR. Specifically, we evaluated two additional types of ASSR measures: the onset slope, and the centroid latency.

3.A. Power onset slope

The power onset slope was estimated using a linear regression model (Supplementary Table 1). In the calculation of the power onset slope, the ‘early period’ was assumed to last until 100 ms. The short-time Fourier transform time window at the terminal point of the early period (i.e., at 100 ms) still covered a portion of the pre-stimulus interval (< 0 ms) since it lasted for -25 ~ 225 ms. Hence, it was reasonable to assume that ASSR measures within the period were under the effect of stimulus onset.

3.B. Power centroid latency

The power centroid latency was defined as a center of gravity as a function of time (Supplementary Table 1). The center of gravity has been commonly regarded as the representative value in various EEG studies, such as event-related potential (ERP) and individual alpha frequency (10, 11). The power centroid latency reflects comprehensive temporal characteristics, including onset and offset effects due to the auditory stimulus. The centroid latency would be relatively delayed provided with some conditions: (i) low gamma response; (ii) slow increase of gamma response after stimulus onset; (iii) slow decrease of gamma response after stimulus offset. The mathematical expressions of the novel ASSR measures are delineated in Supplementary Table 1.

3.C. Mathematical expressions of the additional ASSR measures

**Supplementary** **Table 1**. Additional 40-Hz ASSR measures with their mathematical expressions. Based on two conventional ASSR measures, six additional ASSR measures were derived. ISI-dependent power and ITC were defined as linear trends of the conventional ASSR measures with respect to the ISI conditions. Power and ITC onset slopes were defined as the early period response steepness, during 0 – 100 ms. Power and ITC centroid latencies were defined as gravitational centers of the conventional ASSR measures after the onset of the auditory stimulus (i.e., 0 – 875 ms). It is noted that the onset slopes and centroid latencies yield three values for each ISI condition, different from ISI-dependences.

| ASSR measures | Mathematical expression |
| --- | --- |
| **Total power** |  |
| ISI-dependent power | $P\left( \mathrm{ISI} \right)=b_{1}*\mathrm{ISI}+b_{0}$  (*b_1_*: ISI-dependent power) |
| Power onset slope  (500, 2000, 3500 ms) | $p_{\mathrm{ISI}}\left( t \right)=b_{1, \mathrm{ISI}}*t+b_{0,\mathrm{ISI}}, t\in[0, 100]$  (*b_1_*: power onset slope) |
| Power centroid latency  (500, 2000, 3500 ms) | $T_{centroid, \mathrm{ISI}}=\frac{\int_{0}^{875} t\cdot p_{\mathrm{ISI}}\left( t \right) dt}{\int_{0}^{875} p_{\mathrm{ISI}}(t) dt}$ |
| **ITC** |  |
| ISI-dependent ITC | $\Phi(\mathrm{ISI})=b_{1}*\mathrm{ISI}+b_{0}$  (*b_1_*: ISI-dependent ITC) |
| ITC onset slope  (500, 2000, 3500 ms) | $\phi_{\mathrm{ISI}}\left( t \right)=b_{1, \mathrm{ISI}}*t+b_{0}, t\in[0, 100]$  (*b_1_*: ITC onset slope) |
| ITC centroid latency  (500, 2000, 3500 ms) | $T_{centroid, \mathrm{ISI}}=\frac{\int_{0}^{875} t\cdot\phi_{\mathrm{ISI}}\left( t \right) dt}{\int_{0}^{875} \phi_{\mathrm{ISI}}(t) dt}$ |

ISI, inter-stimulus interval; p (t), the time course of total power; P, total power averaged during the stimulus (0 – 500 ms); b_1_ and b_0_, coefficients for a linear regression model; T*_centroid_*, centroid latency; *ϕ* (t), the time course of inter-trial coherence (ITC); Φ, ITC averaged during the stimulus.

3.D. Phase-locking angle

In addition to the conventional and additional ASSR measures, the phase-locking angle (PLA) was also calculated and analyzed according to Roach et al. (6). For the calculation of the PLA, the complex ITCs were transformed to the phase angles, with real component on the x-axis and imaginary component on the y-axis. Then, the difference between the phase angles for each participant was calculated by subtracting those of ‘expected angles,’ circular-averaged phase angles in HCs for each ISI condition, for each time point. Finally, the phase-locking angles (PLAs) were evaluated by the circular-averaged phase angle difference for 0 – 500 ms. For more detailed information, see Roach et al.’s study (6).

Statistical analyses were conducted on the PLAs, similar to other ASSR measures. For group comparison, the Watson-Williams test was conducted. For multiple comparisons, Bonferroni corrections were applied. The associations between the PLAs and psychotic symptom severity or socio-cognitive function were examined using circular-linear correlation analysis with a bootstrap resampling technique (n = 5,000) to avoid multiple correction issues. The overall circular statistics were calculated following the method described by Berens et al. (12)

**4. Statistical power analysis**

A statistical power analysis was performed using the G*Power program (13). The alpha level was set based on the Bonferroni correction for eight ASSR measures (i.e., 0.05 / 8 = 0.00625). All the other factors were set to their default values. The analysis revealed that a minimum of 32 samples are required, suggesting that the sample size in our study (i.e., 45) was sufficient for the purposes of the study.

**5. Supplementary results: Phase-locking angle**

For all the ISI conditions, patients with SZ showed delayed PLAs compared to HC (Fig. S1). In addition, PLA values in patients with SZ were relatively scattered than those in HCs, resulting in a smaller magnitude of the grand circular-averaged angles. This scattering was particularly evident in the ISI-3500 ms condition.

Patients with SZ showed a significantly delayed PLA in the ISI-500 ms condition (59.77°, *p* = 0.004; Figure S1). This result is consistent with the previous study on PLA by Roach et al. (6). Furthermore, patients with SZ also showed marginally delayed PLA in the ISI-2000 and 3500 ms conditions (40.51°, *p* = 0.063; 57.85°, *p* = 0.085).

The delayed PLA in the ISI-500 ms condition showed a correlated trend with the PANSS positive score (R_c_ = 0.463, p = 0.076; 95%CI 0.047 ~ 0.541; Fig. S2). This result is in line with the previous study reporting an association between phase delay in 40-Hz ASSR and positive symptoms in patients with SZ (14). Furthermore, PLA also might serve as a potentially useful biomarker for diagnosing SZ.


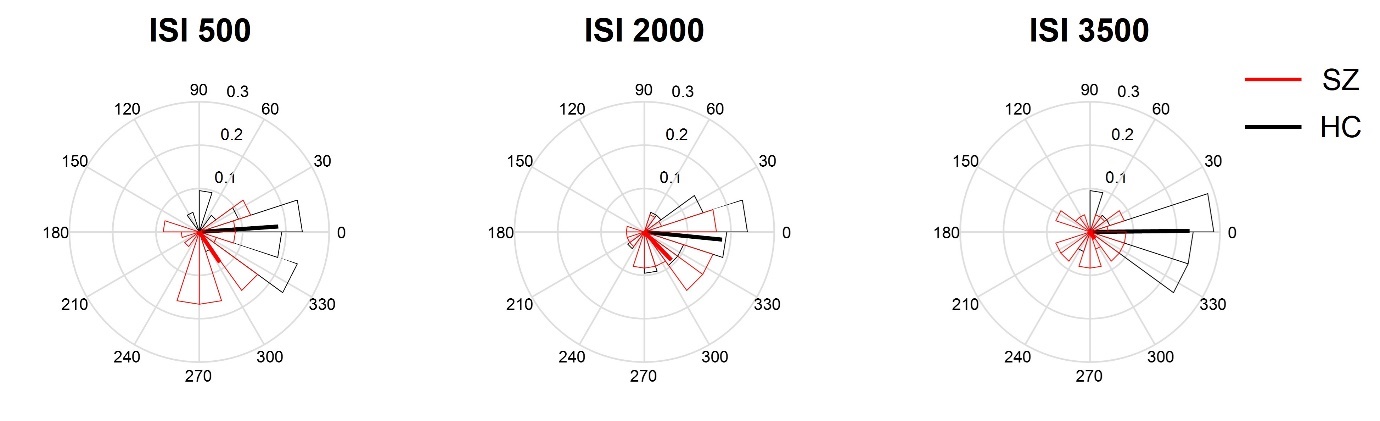


**Figure S1**. Comparison of phase-locking angle (PLA) between patients with SZ and HCs, for each ISI condition. The PLA values of SZ and HC are represented by red and black colors, respectively. The histogram represents the distribution of PLAs, while the solid line indicates the circular-averaged PLAs.


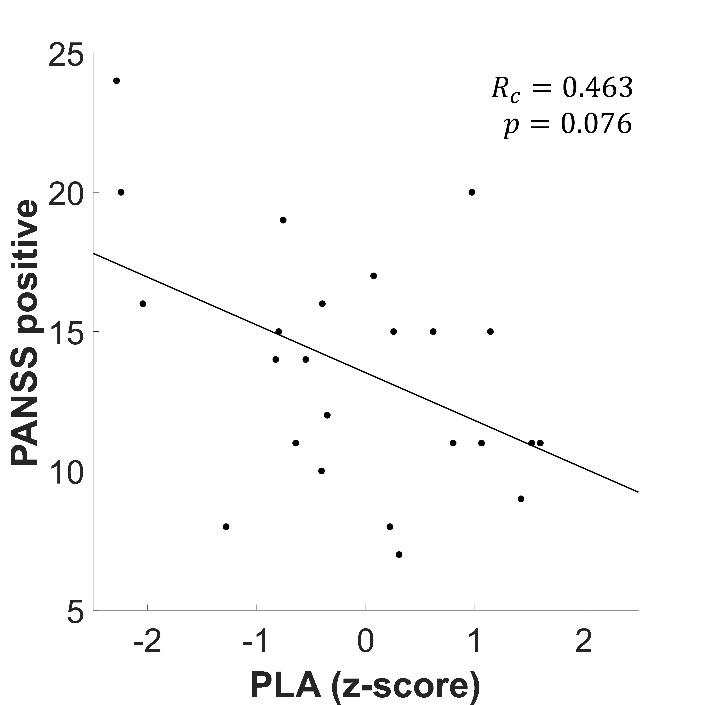


**Figure S2**. Results of circular correlation analysis in patients with SZ. The z-transformed PLA with ISI-500 condition was circular-correlated with the PANSS positive. It is noted that circular-correlation coefficient has positive values only. PLA, phase-locking angle.

**6. References**

1. Association AP (2000): *Diagnostic and statistical manual of mental disorders, 4th edition, text revision (DSM-IV-TR)*. American Psychiatric Association.

2. Rybarczyk B (2011): Social and Occupational Functioning Assessment Scale (SOFAS). In: Kreutzer JS, DeLuca J, Caplan B, editors. *Encyclopedia of Clinical Neuropsychology*. New York, NY: Springer New York, pp 2313-2313.

3. Kiebel SJ, Tallon-Baudry C, Friston KJ (2005): Parametric analysis of oscillatory activity as measured with EEG/MEG. *Hum Brain Mapp*. 26:170-177.

4. Roß, B., Picton, T. W., & Pantev, C. (2002). Temporal integration in the human auditory cortex as represented by the development of the steady-state magnetic field. Hearing research, 165(1-2), 68-84.

5. Tada, M., Nagai, T., Kirihara, K., Koike, S., Suga, M., Araki, T., ... & Kasai, K. (2016). Differential alterations of auditory gamma oscillatory responses between pre-onset high-risk individuals and first-episode schizophrenia. Cerebral cortex, 26(3), 1027-1035.

6. Roach, B. J., Ford, J. M., & Mathalon, D. H. (2019). Gamma band phase delay in schizophrenia. Biological Psychiatry: Cognitive Neuroscience and Neuroimaging, 4(2), 131-139.

7. Griskova-Bulanova, I., Dapsys, K., Melynyte, S., Voicikas, A., Maciulis, V., Andruskevicius, S., & Korostenskaja, M. (2018). 40 Hz auditory steady-state response in schizophrenia: Sensitivity to stimulation type (clicks versus flutter amplitude-modulated tones). Neuroscience Letters, 662, 152-157.

8. Kim, S., Jang, S. K., Kim, D. W., Shim, M., Kim, Y. W., Im, C. H., & Lee, S. H. (2019). Cortical volume and 40-Hz auditory-steady-state responses in patients with schizophrenia and healthy controls. NeuroImage: Clinical, 22, 101732.

9. Kwon, J. S., O'Donnell, B. F., Wallenstein, G. V., Greene, R. W., Hirayasu, Y., Nestor, P. G., ... & McCarley, R. W. (1999). Gamma frequency–range abnormalities to auditory stimulation in schizophrenia. Archives of general psychiatry, 56(11), 1001-1005.

10. Corcoran AW, Alday PM, Schlesewsky M, Bornkessel-Schlesewsky I (2018): Toward a reliable, automated method of individual alpha frequency (IAF) quantification. *Psychophysiology*. 55:e13064.

11. Steinberg J, Truckenbrodt H, Jacobsen T (2012): The role of stimulus cross-splicing in an event-related potentials study. Misleading formant transitions hinder automatic phonological processing. *J Acoust Soc Am*. 131:3120-3140.

12. Berens, P. (2009). CircStat: a MATLAB toolbox for circular statistics. Journal of statistical software, 31, 1-21.

13. Faul, F., Erdfelder, E., Lang, A. G., & Buchner, A. (2007). G* Power 3: A flexible statistical power analysis program for the social, behavioral, and biomedical sciences. Behavior research methods, 39(2), 175-191.

14. Yanagi, M., Tsuchiya, A., Hosomi, F., Terada, T., Ozaki, S., Shirakawa, O., & Hashimoto, M. (2022). Evaluating delay of gamma oscillations in patients with schizophrenia using evoked response audiometry system. Scientific Reports, 12(1), 11327.
